# Myo-Inositol Safety in Pregnancy: From Preimplantation Development to Newborn Animals

**DOI:** 10.1155/2016/2413857

**Published:** 2016-09-06

**Authors:** Nilay Kuşcu, Mariano Bizzarri, Arturo Bevilacqua

**Affiliations:** ^1^Department of Psychology, Section of Neuroscience, Sapienza University of Rome, Via dei Marsi 78, 00185 Rome, Italy; ^2^Department of Experimental Medicine, Sapienza University of Rome, Viale Regina Elena 324, 00161 Rome, Italy; ^3^Research Center in Neurobiology Daniel Bovet (CRiN), 00185 Rome, Italy

## Abstract

Myo-inositol (myo-Ins) has a physiological role in mammalian gametogenesis and embryonic development and a positive clinical impact on human medically assisted reproduction. We have previously shown that mouse embryo exposure to myo-Ins through preimplantation development* in vitro* increases proliferation activity and blastocyst production, representing an improvement in culture conditions. We have herein investigated biochemical mechanisms elicited by myo-Ins in preimplantation embryos and evaluated myo-Ins effects on postimplantation/postnatal development. To this end naturally fertilized embryos were cultured* in vitro* to blastocyst in the presence or absence of myo-Ins and analyzed for activation of the PKB/Akt pathway, known to modulate proliferation/survival cellular processes. In parallel, blastocyst-stage embryos were transferred into pseudopregnant females and allowed to develop to term and until weaning. Results obtained provide evidence that myo-Ins induces cellular pathways involving Akt and show that (a) exposure of preimplantation embryos to myo-Ins increases the number of blastocysts available for uterine transfer and of delivered animals and (b) the developmental patterns of mice obtained from embryos cultured in the presence or absence of myo-Ins, up to three weeks of age, overlap. These data further identify myo-Ins as a possibly important supplement for human preimplantation embryo culture in assisted reproduction technology.

## 1. Myo-Inositol

Myo-inositol (myo-Ins) is the major component of a family of nine hexahydroxycyclohexane inositol (Ins) stereoisomers broadly distributed in eukaryotic tissues and cells, where they are involved in basic biological functions [1].

Ins is incorporated into cell membrane phosphoinositides or phosphatidyl-glycerides as phosphatidyl-inositol (PI), which is phosphorylated by a set of specific phosphoinositide-3-kinases (PI3K) to phosphatidyl-inositol phosphates. Phosphatidyl-inositol trisphosphate (PIP3) can be further metabolized by phospholipase C (PLC) to inositol trisphosphate (Ins−1,4,5P3, InsP3), which acts as second messenger for various hormones such as FSH, TSH, and insulin [2, 3]. Inositol is the precursor of other membrane components and glycosyl-phosphatidyl-inositol (GPI) protein-anchor molecules (reviewed by [4]), signaling molecules (reviewed by [5, 6]), chromatin-remodeling, and transcription-modulating molecules [7]. It finally acts as a hyperosmotic stress protectant [8].

## 2. Myo-Inositol in Reproduction, Gametogenesis, and Embryonic Development

Increasing evidence shows that myo-Ins has a physiological role in mammalian gametogenesis and embryonic development and a positive clinical impact on human medically assisted reproduction.

Together with its isomer D-chiro-inositol (D-chiro-Ins), myo-Ins acts as second messenger of insulin [9] and has been widely studied for its involvement in metabolic syndrome (reviewed by [10]) as well as for the treatment of polycystic ovary syndrome (PCOS) (reviewed by [11, 12]), one of the most common female endocrine disorders [13] strictly associated with insulin resistance [14]. Recent reports strongly suggest that both Ins isomers can be positively associated in the management of PCOS patients enrolled in assisted reproduction procedures [15], with significant encouraging clinical outcomes [16].

While D-chiro-Ins does not appear to have a clear role in the ovary or in the testis, myo-Ins affects gametogenetic and embryogenetic processes at several levels, having a protective, positive role in reproduction and development (reviewed by [17]).

Myo-Ins concentration in the reproductive tracts of male mammals is higher than in blood serum being produced by FSH-responsive Sertoli cells [18, 19]. Studies performed on pathological sperm samples have shown that myo-Ins is crucial in spermatogenesis by positively affecting sperm cell motility [20] and mitochondrial membrane potential [21], an apoptotic marker directly associated with cell viability. Since these parameters are predictors of good embryos quality, the use of myo-Ins in the andrology laboratory is now employed to improve the recovery of sperm cells after swim-up in* in vitro* fertilization (IVF) cycles [20].

As for female gametogenesis, myo-Ins is actively concentrated in mammalian follicular and tubal fluid, being higher than in blood serum [22]. Respective values of myo-Ins concentration in human follicular fluid and serum are 30-to-40 *μ*M [23] and 10-to-20 *μ*M [24]. In addition, a positive correlation exists between follicular myo-Ins content and oocyte quality as well as pregnancy outcome [23].

At the ovarian level, Myo-Ins has different functions in both follicle cells and oocytes. However, although it is known that myo-Ins metabolism is severely deregulated in follicle cells of PCOS patients [25], effects of this molecule on somatic ovarian cells still need to be evaluated. We have hypothesized that in theca and granulosa cells myo-Ins may sustain steroidogenic activity during the ovarian cycle by modulating the dynamics of cytoskeletal structures [26]. Research on this issue is ongoing in our laboratories.

Myo-Ins is transported into mammalian cells, including growing and fully-grown oocytes and early preimplantation embryo blastomeres, by a sodium cotransporter [27] and a sodium independent transporter [28]. In oocytes, activity of myo-Ins−derived InsP3 on the modulation of intracellular Ca^2+^ concentration in response to the action of the hormones LH and FSH [29, 30] is well known. It acts via cell-specific receptors (InsP3-R1) [31] and appears to play a key role in meiotic maturation [32].

Chiu et al. [33] cultured preovulatory oocytes from outbred mice in minimal essential medium supplemented with myo-Ins and observed increased rates of maturation, IVF, and development to the two-cell stage with respect to oocytes cultured in medium alone, suggesting that higher availability of myo-inositol increases both meiotic and activation efficiencies of the oocyte. After transfer to foster mothers, the implantation rate and postimplantation viability of these embryos were also increased in the myo-Ins treated groups [33].

During mammalian preimplantation development, activity of myo-Ins transporters ensures a robust uptake, which progressively increases from the one-cell to the blastocyst stage [34, 35], suggesting a parallel increase in cellular requirement of the molecule. It has been shown that, in preimplantation embryo blastomeres, myo-Ins is rapidly incorporated into phosphoinositides [34], leading to raised intracellular InsP3 levels. This led several groups, including ours, to investigate whether myo-Ins could also have a positive action during preimplantation development in both the laboratory mouse and farming species.

Myo-Ins supplementation of culture media was found to improve rabbit and bovine blastocyst formation and expansion [35, 36]. Culture of rabbit embryos at the morula stage in medium containing myo-Ins at the optimal concentration of 75 *μ*M resulted in blastocysts expansion with a fourfold increase in diameter when compared to that provided by standard culture conditions [35]. Similar observations were obtained on bovine zygotes matured and fertilized* in vitro* [36], after culture in* synthetic oviduct fluid* medium [37] in the presence or absence of 2.77 mM myo-Ins. As a result, blastocyst rate was higher among embryos developed in the presence of myo-Ins [36]. To evaluate postimplantation effects of this treatment, ten blastocysts grown in the presence of myo-Ins were transferred to foster cows and developed to term, producing five healthy animals [36].

**Figure 1:**
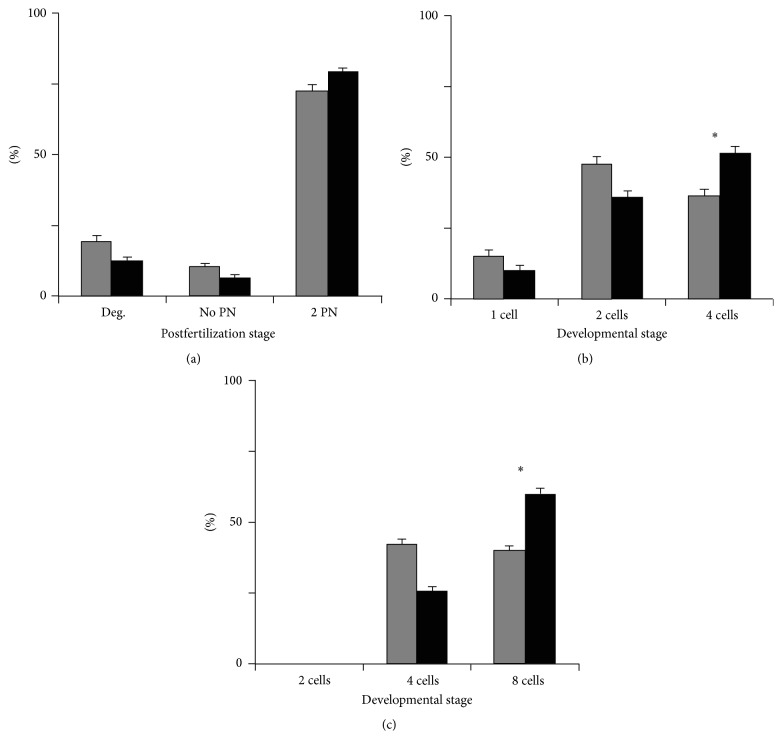
Effect of myo-inositol medium supplementation on pronuclear formation and embryo development during the first two days of* in vitro* culture. Completion of early developmental steps by zygotes cultured in the absence (grey bars) or the presence (solid bars) of 10 mM myo-inositol. Zygotes were scored 6 hours (a) and embryos at 24–26 hours (b) and 48–50 hours (c) p.f. Bars represent the mean ± SEM of 7 independent experiments of the fraction of embryos at each indicated stage. (a) N, myo-Ins− embryos (grey bars), 121; myo-Ins+ embryos (solid bars), 123. ((b), (c)) N, myo-Ins− embryos (grey bars), 97; myo-Ins+ embryos (solid bars), 106. Asterisks indicate difference between treatments calculated by *χ* ^2^ test with Yates correction for continuity: (b) *P* < 0.05; (c) *P* < 0.01.

**Figure 2:**
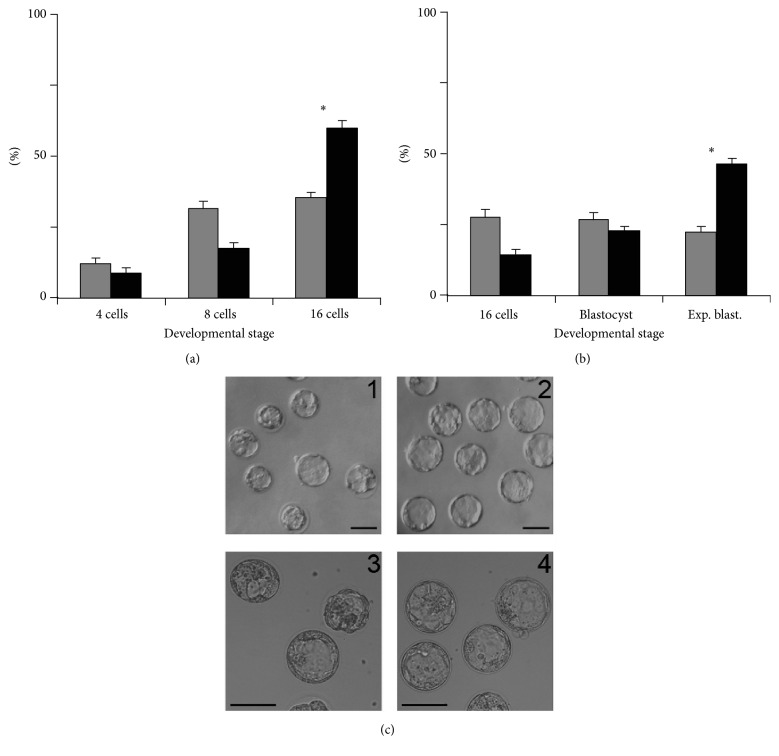
Effect of myo-inositol medium supplementation on embryo development during the third and fourth days of* in vitro* culture. Completion of mid-to-late preimplantation development by embryos cultured in the absence (grey bars) or the presence (solid bars) of 10 mM myo-inositol. Embryos were scored 72–74 hours (a) and 96–98 hours (b) p.f. Bars represent the mean ± SEM of 7 independent experiments of the fraction of embryos at each indicated stage. ((a), (b)) N, myo-Ins− embryos (grey bars), 97; myo-Ins+ embryos (solid bars), 106. Exp. blast.: expanded blastocyst. Asterisks indicate difference between treatments calculated by *χ* ^2^ test with Yates correction for continuity: (a) *P* < 0.05; (b) *P* < 0.05. (c) Representative images of expanded and nonexpanded blastocysts observed at day 4 among myo-Ins− (1, 3) and myo-Ins+ (2, 4) embryos; magnification: 40x (1, 2); 80x (3, 4). Black bars, 100 *μ*m. Panel (b) is modified from [38].

With a clear interest in human assisted reproduction technology (ART), positive data on myo-Ins in mammalian preimplantation development prompted us to hypothesize that inclusion of myo-Ins in embryo culture media would result in increased numbers of high quality embryos produced by IVF/intracytoplasmic sperm injection (ICSI). We tested this hypothesis in a previous work [38], using the mouse embryo model [39, 40], by investigating the effects of myo-Ins supplementation of sequential human embryo culture media, starting 30 minutes after fertilization (p.f.) and for the whole length of preimplantation development (day 4 p.f.). In that study, we cultured embryos obtained by ICSI from gametes of inbred C57BL/6N mice, in which ICSI results in suboptimal rates of oocyte survival and blastocyst development, when compared to hybrid mice [41]. After fertilization in unmodified fertilization medium, embryos were cultured in cleavage medium in the presence or absence of 10 mM myo-Ins (myo-Ins+ and myo-Ins−, resp.) and monitored daily for developmental progression. At all-time intervals monitored, (p.f.), myo-Ins+ embryos displayed a faster cleavage rate, with a higher percentage of embryos at the most advanced stage. In these embryos, early differentiative events such as compaction and blastulation occurred at the proper developmental stage, excluding apparent toxic effects of myo-Ins. Although results scored on day 4 have already been published [38], those recorded at intermediate times have not been presented elsewhere and are reported in Figures 1 and 2, together with embryo morphology observed on day 4.

Embryos cultured in the presence of myo-Ins were developmentally advanced with respect to control embryos, being mostly represented by expanded blastocysts with a higher number of blastomeres, as shown by Hoechst 33343 nuclear staining [38]. We concluded that myo-Ins supplementation represents an improvement of culture conditions reducing the developmental gap typically observed between embryos obtained and cultured* in vitro* and those developed* in vivo,* further supporting its possible use for human embryo preimplantation culture.

One of the issues left uncovered by these experiments concerns the nature of biochemical pathways induced by exposure to myo-Ins in preimplantation of embryo blastomeres. Existing information suggests that possible pathway(s) elicited by myo-Ins in mouse embryo blastomeres and responsible for a reduced length of cell cycles and a more efficient proliferation activity are initiated via rapid incorporation into phosphoinositides [34], namely, PIP3, enzymatically fostered by activity of PI3K. In turn, PIP3 is converted by PLC into diacylglycerol and InsP3, which, by mobilizing Ca^2+^ stores, has already been recognized to speed up several cellular processes, including proliferation [42, 43]. PI3K is also responsible for the activation of the PKB/Akt pathway [44], which is known to promote proliferation of mouse embryo blastomeres [45]. As myo-Ins has been shown to increase Akt phosphorylation and hence its activity in mouse skeletal muscle cells [46], we hypothesized that a critical step in enhancing embryo preimplantation development may be represented by Akt activation induced by myo-Ins supplementation [47]. We here provide the first evidence, obtained by immunofluorescence analysis, that myo-Ins supplementation of culture media increases serine phosphorylation of PKB/Akt in mouse preimplantation embryos.

A second open issue in current research concerns the safety of preimplantation embryo exposure to myo-Ins for postimplantation and postnatal development.

Besides the reported observations on bovine embryos [36], it could be argued that preimplantation embryos, including human embryos, were once routinely cultured in undefined media supplemented with commercial BSA preparations or batches of bovine or human sera, which contain variable amounts of myo-inositol, without reporting any apparent negative effect.

In addition, myo-Ins appears important for normal development (reviewed by [17]). Its concentration in foetal human serum is severalfold higher than in adults, lowering only around birth [48], and its administration during pregnancy has positive effects on pathological conditions in both humans [49, 50] and rodent species [51–54].

All data hitherto reported suggest that preimplantation embryo exposure to myo-Ins would have no detrimental effects on further development. To obtain more direct information on this issue, we transferred blastocysts produced* in vitro* in the presence of myo-Ins into recipient foster mothers and allowed their development to term. By this approach, we obtained healthy offspring that appeared normal in the sex ratio and, at least until weaning, somatometrically. These experiments provide additional data on myo-Ins effects on mammalian preimplantation embryos and strongly suggest that it can be considered safe for embryo development to term.

## 3. Materials and Methods

### 3.1. Animals

Animals (Charles River Italia, Calco, VA, Italy) were housed in a temperature-controlled facility (22 ± 1°C) on a 12/12 h light/dark cycle, inside standard cages with unlimited access to food and water. Forty-to-60-day-old C57BL/6N mice were used as donors of oocytes and male studs. Forty-to-60-day-old CD/1 mice were used as foster mothers and vasectomized male studs. All experimental procedures were conducted in accordance with the official European guidelines for the care and use of laboratory animals (86/609/EEC). Experimental protocols and related procedures were approved by the Italian Ministry of Public Health.

### 3.2. Culture Media

In order to model human preimplantation embryo culture, all procedures were performed in commercial Quinn's Advantage media and supplements (BioCare Europe, Napoli, Italia), which do not contain myo-Ins, as described [38].

### 3.3. Animal Treatment and Zygote Collection

Female mice were hormonally induced by intraperitoneal injections of 5 IU pregnant mare serum gonadotropin (NHPP, Torrence CA, USA) and, 46–48 h later, of 5 IU human chorionic gonadotropin (Corulon, Intervet Italia, Aprilia, LT, Italy). After mating with male mice ON, females carrying a vaginal plug were sacrificed by cervical dislocation and cumulus-oocyte complexes were collected from the oviducts, freed from cumulus cells by treatment with 0.1% hyaluronidase in Hepes buffered Quinn's Protein Plus Advantage HTF-Medium containing 5% human serum albumin, rinsed in HTF-Medium, and incubated at 37°C in Quinn's Protein Plus Cleavage Medium (C-Medium) under a humidified atmosphere of 5% CO_2_ in air.

### 3.4. Embryo Culture

One-cell embryos were positively scored by the presence of two pronuclei and then divided into two groups. One group was further cultured in 14 *μ*L/mL myo-inositol (10 mM, Andrositol® LAB Lo.Li. Pharma, Italy) in C-Medium and the other one in 14 *μ*L/mL phosphate buffered saline in C-Medium. Embryo culture was performed in 500 *μ*L medium inside Falcon 4 well IVF plates without medium replacement, as described [38]. Developing embryos were scored daily for morphology and progression through cleavage stages.

### 3.5. Detection of Phosphorylated PKB/Akt

Preimplantation embryos at the morula or blastocyst stage were washed in 0.1 M phosphate buffered saline, pH 7.4 (PBS), containing 0.3% bovine serum albumin (BSA) (PBS-BSA), and were then fixed in 4% paraformaldehyde in PBS for 30 min at room temperature (RT). After three washes in PBS-BSA, embryos were permeabilized in PBS containing 0.25% Tween-20 (Sigma; St. Louis, MO) for 15 min at RT and washed three times in PBS-BSA. Permeabilized embryos were processed for immunostaining by an overnight incubation at 4°C in the presence of the primary antibody, rabbit monoclonal anti-phospho-Akt (Ser473, #4080), anti-phospho-Akt (Thr308, #4056) or anti-Akt (total, #9272) antibody (Cell Signaling Technology, Beverly, MA, USA) (1 : 75), followed by washing in PBS-BSA and 1 hr final incubation with the secondary, and Alexa Flour-488 conjugated secondary antibodies (Invitrogen), diluted 1 : 200 in PBS-BSA for 1 h at RT. After washing with PBS-BSA, blastomere nuclei were counterstained with 1 mg/mL Hoechst 33343 for 10 min at RT. Finally, embryos were washed three times for 10 min each in PBS-BSA and mounted in PBS and glycerol (1 : 1 v/v) using coverslips. Fluorescent signals were detected using a Zeiss AxioPlan fluorescence microscope (Carl Zeiss, Oberkochen, Germany) at 400x magnification. For semiquantitative analysis of fluorescence, embryos at various developmental stages were immunostained after pooling in the same drops. Fluorescence emission was collected under similar excitation conditions and then quantitatively analyzed by using the ImageJ software (ImageJ 1.47v, Wayne Rasband, National Institutes of Health, USA. http://imagej.nih.gov/ij/).

### 3.6. Embryo Transfer and Development to Term

In ten replicate experiments, embryos developed to the blastocyst stage after 4 days of culture in myo-Ins+ C-Medium or myo-Ins− C-Medium were transferred to the uteri of pseudopregnant foster mothers mated 2.5 days earlier with vasectomized males and carried to term [55]. On the day of delivery, newborn animals were weighed, checked for gross abnormalities, and left to be nursed by their moms until weaning. Preweaning morphological analyses included body growth at one week, fur appearance, and eye opening. Mice were finally weighed and sexed at weaning (three weeks of age).

### 3.7. Chemicals

Where not stated otherwise, chemicals were purchased from Sigma-Aldrich Co. (St. Louis, MO, USA).

### 3.8. Data Analysis

Data were analyzed by using Student's *t*-test or *χ* ^2^ tests with Yates continuity correction. Statistical analyses were performed using R: A language and environment for statistical computing (R development core team, R foundation for statistical computing, ISBN 3-900051-07-0, 2008, Vienna, Austria. https://www.r-project.org/).

## 4. Results

### 4.1. Analysis of Myo-Ins−Dependent Akt Phosphorylation

**Figure 3:**
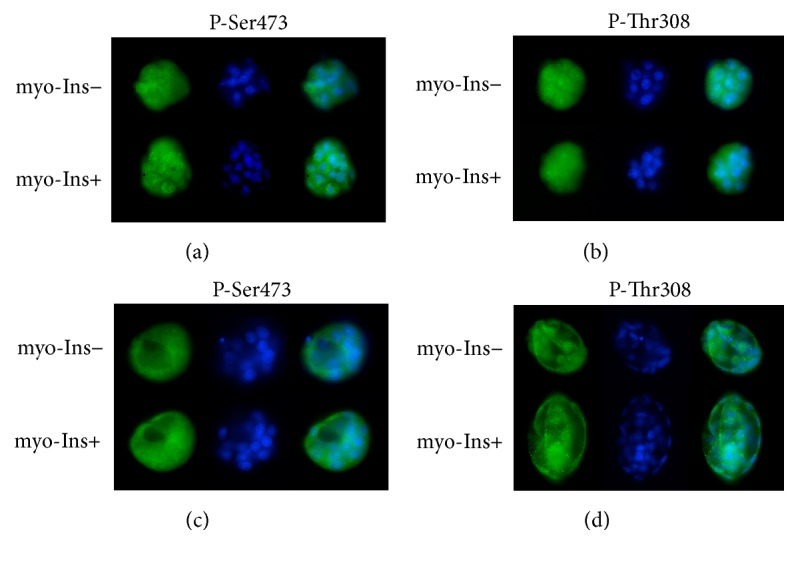
Localization of phosphorylated Akt in preimplantation embryos at the morula and blastocyst stage, after continuous culture in the presence or absence of myo-Ins. ((a), (b)) morula stage embryos; ((c), (d)) blastocyst stage embryos. In each panel, from left to right: FITC, Hoechst 33343, merge. The same pattern was consistently observed in all embryos analyzed; panels show representative embryos. Statistical analysis on these embryos, reported in the text, was performed on at least three embryos at the morula and blastocyst stages in three independent experiments.

We have determined presence and phosphorylation of Akt in late preimplantation stage embryos cultured in the presence or absence of myo-Ins by immunofluorescence analysis. This approach revealed the presence of serine 473- and threonine 308-phosphorylated Akt in both morula and blastocyst embryos (Figure 3). Phosphorylated Akt was localized prominently in blastomere cytosols but a limited nuclear localization was also observed. The relative content of phosphorylated Akt at the same stages was also measured by quantification of immunofluorescence data (Table 1). The level of serine 473-phosphorylated Akt did not appear to be modified in embryos cultured in the presence of myo-Ins at the morula stage (*t*4 = 1.75, *P* = 0.15) (Figure 3(a)) but it was increased at the blastocyst stage (*t*4 = 3.61, *P* = 0.02) (Figure 3(c)). On the contrary, the level of threonine 308-phosphorylated Akt did not vary significantly depending on the presence or absence of myo-Ins (morulae, *t*4 = 0.670, *P* = 0.54, Figure 3(b); blastocysts, *t*4 = 0.640, *P* = 0.56, Figure 3(d)). Amounts of total Akt did not vary under different conditions (not shown).

### 4.2. Effects of Preimplantation Embryo Exposure to Myo-Ins on Development to Term

In ten replicate experiments, all embryos that had developed* in vitro* to the expanded or nonexpanded blastocyst stage in the presence or absence of myo-Ins were transferred to foster mothers and allowed to develop through birth and until weaning. Of 105 myo-Ins+ and 76 myo-Ins− blastocysts transferred, 59 (56.2%) and 33 (43.4%), respectively, were delivered (*χ* ^2^ = 2.876, *P* = 0.09). Although this difference was not significant, when we compared numbers of delivered animals with numbers of fertilized oocytes cultured under the two conditions, 154 myo-Ins+ one-cell embryos and 147 myo-Ins− one-cell embryos, we obtained a significant improvement in the overall efficiency of the treatment (*χ* ^2^ = 8.91, *P* = 0.003). A similar difference was observed by comparing numbers of fertilized oocytes with numbers of transferred embryos (*χ* ^2^ = 8.52, *P* = 0.003). Overall results are shown in Table 2.

Body weights at birth were 1.42 ± 0.01 g for myo-Ins+ and 1.41 ± 0.02 g for myo-Ins− (*t*90 = 0.645, *P* = 0.52). During the first week, six and four pups were found dead in the myo-Ins+ and myo-Ins− groups, respectively. Somatometric development appeared similar in mice of both groups, with appropriate acquisition of body fur and eye opening. Weights at one week of age were also similar (myo-Ins+, 2.06 ± 0.16 g, myo-Ins−, 2.08 ± 0.19 g; *t*80 = 0.286, *P* = 0.78). Sex distributions in the two conditions were both casual (myo-Ins+, 27 males, 26 females, myo-Ins−, 14 males, 15 females). Finally, weights at three weeks of age were similar for sexes and embryo culture conditions (males: myo-Ins+, 10.20 ± 0.21 g; myo-Ins−, 10.29 ± 0.28 g, *t*39 = 0.790, *P* = 0.43; females: myo-Ins+, 9.90 ± 0.19 g; myo-Ins−, 9.75 ± 0.40 g, *t*39 = 1.214, *P* = 0.23).

## 5. Discussion

Akt represents a major downstream effector of growth factors, cytokines, and adhesion receptors capable of promoting cell survival and proliferation [56, 57] and is regulated, among others, by PIP3, the second messenger product of PI3K, via recruitment of PDK1. PDK1 induces direct phosphorylation at threonine 308 [58] and appears to be also implicated in the negative regulation of phosphorylation at serine 473, by direct binding to this hydrophobic site. However, PDK1 can be displaced by an appropriate signal [59, 60], including activation of integrin Like Kinase (ILK) [44], allowing Akt autophosphorylation or phosphorylation by other kinases at serine 473 [58]. This pathway makes Akt phosphorylation of serine 473 inducible by upstream signals; on the contrary, phosphorylation of threonine 308 appears to have a constitutive nature [56].

We have previously shown that, during the first two stages of embryo development, Akt phosphorylation is not prevented by inhibition of either PI3K or PDK1 and concluded that the phosphorylation state and the intracellular distribution of Akt in two-cell embryos are independent of the activity of both kinases. This suggested that Akt is inherited from the oocyte in its phosphorylated/dephosphorylated form at both serine 473 and threonine 308 [45].

We now show by quantitative immunofluorescence analysis that serine 473 phosphorylation of Akt can be increased in late preimplantation embryos by the presence of myo-Ins in the culture medium. It thus appears that, after the initial stages of development, new phosphorylation of Akt may occur in mid-to-late preimplantation stages depending on availability of myo-Ins. In agreement with previous observations that development of mouse preimplantation embryos requires PI3K activity from the 8/16-cell stage [45], it would be reasonable to hypothesize that this finding can be ascribed to the increased production of phosphoinositides [34] and consequent increased production of PIP3 by PI3K. An increase in phosphorylation of Akt may be responsible for the faster developmental rate of embryos cultured in the presence of myo-Ins.

The lack of inducibility of threonine phosphorylation of Akt by myo-Ins is also in agreement with previous observations [56].

It is puzzling that myo-Ins exerts opposite effects on both PI3K and Akt activities when added to cancer cell cultures [61], in which it has been found to reduce PI3K levels as well as Akt activity by inhibiting its phosphorylation. To this respect, it is therefore tempting to speculate that such effect could be considered context-dependent and that some other still unknown factors are likely to participate in modulating the PI3K/Akt pathway. In order to obtain more specific information on this pathway, we are currently analyzing the effects of myo-Ins both on development and on Akt phosphorylation in preimplantation embryo in the presence of inhibitors of PI3K and PDK1 [45]. Further experiments will address the involvement on proliferative activity of preimplantation embryos blastomeres of pro- and anti-apoptotic factors of the Bcl-2 family [62].

Data here produced represent a first assessment of the effect of preimplantation embryo exposure to myo-Ins on mouse development to term.

So far, information on this issue in mammals is limited to one finding obtained on bovine embryos cultured in the presence of 2.77 mM myo-Ins [36]. In that study no comparison was made between embryos cultured in the two conditions, but blastocysts that had developed after preimplantation exposure to myo-Ins were transferred producing healthy animals.

Present results obtained in the mouse show (a) the apparent absence of early toxic effects of myo-Ins, as suggested by normal prenatal and short-term postnatal development, and (b) a significant increase in the overall rate of live births obtained after preimplantation embryo culture in myo-Ins and subsequent transfer into foster mothers.

If the first observation was expected in light of the body of information here reported on the positive effects displayed by myo-Ins on mammalian gametogenesis and development, the second one deserves particular attention. In fact, it supports the possibility that a regular use of myo-Ins as culture supplement provides high efficiency in the production of viable preimplantation embryo* in vitro* both in the mouse and in farming specie with promising outcome for both scientific and economic purposes.

In addition, it strengthens the hypothesis that the use of myo-Ins would have a similar positive role in the culture of* in vitro* produced human embryos, with obvious medical consequences.

To this end, however, additional assessments of myo-Ins effects are necessary at least at three different levels [63]: (a) on the expression of imprinted genes during development; (b) on the acquisition of sensory/motor/behavioral functions during early development; and (c) on long-term consequences on the whole organism.

## Figures and Tables

**Table 1:** Effect of myo-Ins on Akt phosphorylation of late preimplantation mouse embryos.

	Morulae		Blastocysts	
	Ser473	Thr308	Ser473	Thr308
Myo-Ins+	65.41 ± 5.01	47.77 ± 4.37	49.33 ± 6.16	32.15 ± 1.56
Myo-Ins−	59.80 ± 2.55	45.87 ± 2.23	38.32 ± 2.54^a^	33.09 ± 2.03

Data represent arbitrary fluorescence units (mean ± SD) of embryos at the stages indicated, pooled from three replicate experiments.

^a^ *P* = 0.02, calculated by Student's *t*-test.

**Table 2:** Mouse development after IVF and exposure of preimplantation embryos to myo-Ins.

	Number of fertilized oocytes	Number of transferred blastocysts (%)	Number of delivered animals (%)
Myo-Ins+	154	105 (68.2)	59 (38.3)
Myo-Ins−	147	76 (51.7)^a^	33 (22.5)^a^

Data were pooled from ten replicate experiments.

Superscripts indicate differences between myo-Ins+ and myo-Ins− embryos (*P* < 0.05) calculated by *χ* ^2^ test; differences are referred to fertilized oocytes.
